# Young Workers’ Access to and Awareness of Occupational Safety and Health Services: Age-Differences and Possible Drivers in a Large Survey of Employees in Italy

**DOI:** 10.3390/ijerph15071511

**Published:** 2018-07-17

**Authors:** Nico Dragano, Claudio Barbaranelli, Marvin Reuter, Morten Wahrendorf, Brad Wright, Matteo Ronchetti, Giuliana Buresti, Cristina Di Tecco, Sergio Iavicoli

**Affiliations:** 1 Institute of Medical Sociology, Centre for Health and Society, Medical Faculty, University of Duesseldorf, 40225 Duesseldorf, Germany; marvin.reuter@uni-duesseldorf.de (M.R.); wahrendorf@uni-duesseldorf.de (M.W.); 2 Department of Psychology, Sapienza University of Rome, 00185 Rome, Italy; Claudio.barbaranelli@uniroma1.it; 3 School of Psychology, & Public Health, La Trobe University, Melbourne 3086, Australia; b.wright@latrobe.edu.au; 4 Italian Workers’ Compensation Authority (INAIL), Department of Occupational and Environmental Medicine, Epidemiology and Hygiene, Monte Porzio Catone, 00078 Rome, Italy; m.ronchetti@inail.it (M.R.); g.buresti@inail.it (G.B.); c.ditecco@inail.it (C.D.T.); s.iavicoli@inail.it (S.I.)

**Keywords:** young workers, occupational safety and health services, occupational health, age-differences, awareness, access to OSH services

## Abstract

Young workers are in particular need of occupational safety and health (OSH) services, but it is unclear whether they have the necessary access to such services. We compared young with older workers in terms of the access to and awareness of OSH services, and examined if differences in employment conditions accounted for age-differences. We used survey data from Italy (INSuLA 1, 2014), with a sample of 8000 employed men and women aged 19 to 65 years, including 732 young workers aged under 30 years. Six questions measured access to services, and five questions assessed awareness of different OSH issues. Several employment conditions were included. Analyses revealed that young workers had less access and a lower awareness of OSH issues compared with older workers. For instance, odds ratios (OR) suggest that young workers had a 1.44 times higher likelihood [95%—confidence interval 1.21–1.70] of having no access to an occupational physician, and were more likely (2.22 [1.39–3.38]) to be unaware of legal OSH frameworks. Adjustment for selected employment conditions (company size, temporary contract) substantially reduced OR’s, indicating that these conditions contribute to differences between older and younger workers. We conclude that OSH management should pay particular attention to young workers in general and, to young workers in precarious employment, and working in small companies in particular.

## 1. Introduction

Young workers are in particular need of occupational safety and health (OSH) services [1,2]. It is well documented, for example, that injury rates are significantly higher among young workers than among older and more experienced workers [3,4,5]. To effectively protect young workers against injuries and other work related diseases, special consideration must be given to improving access to core OSH services, and using safety training for young workers to increase their knowledge and compliance [4,6,7]. Political awareness of this issue is considerable and prompted the European Healthy Workplaces Campaign 2016–2017 to specifically focus on promoting healthy work practices for new employees. This points to the crucial role of life-course prevention [8].

A focus on young workers is a well justified political objective, but this requires that OSH programs are equally effective at protecting both young and older workers [6,9]. This does not necessarily seem to be the case. Although few studies have investigated age differences of OSH quality, findings suggest that young workers may be systematically disadvantaged in terms of the access to and quality of core OSH services [10,11]. Such age-differences may point to structural disadvantages that go beyond the common phenomenon that an inevitable lack of on-the-job experience in young workers correlates with a lower awareness about occupational risks and available OSH services [7,12]. Instead, it can be hypothesised that the comparably weak position of young people in regard to employment conditions and power relations at work is associated with lower standards of OSH services and protective measures for the young [13,14]. This hypothesis refers to recent theories about the interrelatedness of macro-level policies, meso-level structures on the level of local labour markets or single companies, and individual-level experiences of adverse working conditions and a lack of occupational safety and health protection [15,16,17,18].

Precarious employment conditions, such as short-term employment contracts or low-wage work [19,20], are disproportionally more prevalent amongst young workers across Europe and beyond. Across the EU-27 countries in 2016, for example, the proportion of young employees with temporary contracts in the EU-27 was almost three times higher than the proportion in the general working population (14.2% vs. 43.7%) [21]. Precarious or insecure employment conditions are not only independent risk factors for somatic and mental disorders, but seem to be related to lower standards of OSH protection [22,23,24,25,26,27,28,29,30]. One possible explanation is that young professionals may be ready to accept unfavourable working conditions and a lack of protection to gain or to maintain a job in times of high youth unemployment [11,14,22]. Moreover, they may be more reluctant to ask for better and safer working conditions [2]. It can also be hypothesised that employers put less emphasis on implementing OSH management and associated training for temporary workers [26,31,32,33]. Such associations may be further aggravated by the high youth unemployment rate in many countries. Job starters in countries, like Greece, Italy, or South Africa, face youth unemployment rates considerably higher than 30% [34]. High unemployment rates have severe consequences for job seekers, but also impact those who are actively employed as the consequences of job loss are more anxiety-provoking when re-employment is unlikely.

Age-related segregation also affects access to certain types of employment. Young people, especially in economies with high youth unemployment rates, such as Italy or Greece, have little opportunity to gain employment in public sector jobs or occupations with larger companies with higher OSH standards. Instead, they are overrepresented in occupations in construction work, elementary occupations, or in small businesses where OSH management is often less developed than in larger companies [1,2,3,35,36,37].

In sum, there are reasons to assume that age-differences in OSH service access and quality exist and that they are, at least partly, explained by the specific employment conditions of young workers in contemporary economies. Yet, there is a striking lack of theoretical literature and empirical research on the relationship between OSH service provision and worker age [4,13]. Therefore, this study aims to compare the access and awareness to different types of OSH services between younger and older workers. A second objective is to investigate whether different employment conditions, like precarious employment or company size, account for age-differences in OSH access and awareness. Two respective hypotheses guided our analytical approach:

**Hypothesis** **1.** *Young workers have less access to occupational safety and health services, and are less aware of OSH issues compared to older workers.*

**Hypothesis** **2.** *If age-differences in access and awareness exist, they are partly explained by a selection of young workers into occupations and employment conditions that are related to lower OSH management standards, such as precarious employment.*

We will study the hypotheses in a representative sample of Italian workers, a country particularly affected by the recent financial crisis and with a high rate of youth unemployment.

## 2. Materials and Methods

### 2.1. Data Source

We used data from a sample (INSuLA 1) of 8000 employed Italian men and women from 2014, which has been conducted by the Italian workers compensation authority (INAIL). The cross-sectional survey was conducted in collaboration with TNS Italia, who carried out the interviews, and with the National Institute for Statistics (ISTAT), who assisted in calculating the strata/quota for the sampling. The aim of the INSuLA 1 survey was to collect comprehensive information on issues of occupational safety and health at work, including working and employment conditions and perceptions of occupational safety and health services [38,39].

A quota sampling was applied. The source population were all workers in Italy employed for at least 2 months for at least an hour a week in the last 6 months, and whose employment is regulated by the national legal framework for health and safety (Legislative Decree 81/08; excluding all occupations with specific regulations, such as the armed forces or civil protection volunteers). Since there was a lack of official national data, ISTAT extracted representative data of around 17,000 workers from their last national workforce survey [40]. This information was used to define the strata and their quota for the following characteristics: Regional distribution, gender, age, type of contract, occupational level, and economic sector.

A random sample (random digit dial) was drawn and respondents were classified according to the stratification characteristics. Sampling was continued until the fixed proportion of each strata was reached. The final sample consisted of 8000 workers (item non-response is quantified in the respective tables). The distribution of the strata characteristics matched the pre-set quota well, however, weighting was applied to reach the proportion of the source population exactly.

Data was collected using a standardized questionnaire. The interview was carried out by trained interviewers using the Computer Assisted Telephone Interview (CATI) methodology. The questionnaire was tested through a pilot study to check its compatibility with the CATI methodology in regard to the duration of the interview and clarity of the questions.

### 2.2. Measures

Dependent variables: We measured self-reported access to OSH services and awareness about OSH with several indicators. Access to OSH services was measured with six items. Thereby, five items assessed the availability of: (a) An environmental health and safety manager at the workplace, (b) an occupational physician, (c) an occupational health and safety workers’ representative, (d) any OSH related training programs provided by the company in the past 5 years, and (e) any information about health and safety provided by the company in the past five years. Workers who reported they had no access or did not know if the respective service was provided were defined as having no access. Additionally, we recorded if the respondent had a least one medical examination by the occupational physician in case this service was provided in the company. Awareness about OSH norms was measured by five items: (a) Awareness of the existence of the national health and safety regulatory framework, (b) awareness of fire and emergency procedures, (c) awareness about basic first aid procedures, (d) awareness of the legal obligations of the employee for OSH, and (e) an autonomous search for OSH information by the respondent (e.g., internet search). All items were coded using a dichotomous (yes/no) scale to indicate an awareness/lack of awareness of the respective topic.

Covariates: The following employment information was also collected to assess if these factors impacted on OSH service availability and awareness: As an indicator of occupational position we ascertained whether participants had a manual or non-manual job, and an additional category for those in vocational training or others. In addition, the type of contract, mean weekly working hours, economic sector, and size of the company were included. Details on each variable and used categories are shown in Table 1. Additionally, we collected data on the gender and highest educational degree (low, medium, high) of the respondents.

### 2.3. Statistics

The aim of this study is mainly explorative, and we first applied bivariate statistics to compare OHS services between young (<30 years) and older (30+ years) workers. Specifically, we calculated the prevalence of each OSH service in the two age groups, including tests of significance (*p*-values based on chi-square tests) (Hypothesis 1). Although young workers are often defined as workers younger than 25 years we used a broader age span of 19–29 years as previously recommended by other initiatives [41]. The main rationale for choosing this cut-off is the high youth unemployment rate in Italy, which provokes a comparably high age of labour market entry and a low labour force participation of particular young people. Additionally, multivariable logistic regression models were calculated to assess the magnitude of age (independent variable) differences before and after adjustment for the covariates gender (when analysing the complete sample) and education for each OSH indicator (independent variable). Regression models were then additionally adjusted for the employment related variables of occupational position, type of contract, working hours, economic sector, and company size. This adjustment set was applied to assess if associations between OSH indicators and age were influenced by age differences in these employment conditions (Hypothesis 2). All calculations were conducted for the total sample and for men and women separately (results not shown). All calculations were conducted with IBM SPSS version 25 (IBM, Armonk, NY, USA).

## 3. Results

### 3.1. Sample Description

Of the 8000 employed men and women, 732 were under 30 years old (Table 1). Men were slightly overrepresented in the sample aged 30 and older (53.1% men), and this gender imbalance was even higher among young workers (60.8% males).

The employment conditions of young workers differed markedly from the older age group. Manual jobs were more common among the young and their working hours were longer. Young workers were overrepresented in the construction, trade, hotel, and catering sector, while they were significantly underrepresented in public employer dominated sectors, including health, social work, education, and public administration. Pronounced differences exist in regard to employment contract conditions. The proportion with non-permanent contracts was 41.5% for the young compared with only 12.5% in older workers. A clear age gradient was also present for the company size, with a higher proportion of young people working in small companies. Gender specific analyses showed that the relative differences in employment conditions between the age groups were largely similar for men and for women, although men worked more often in manual occupations in industry, construction, and manufacturing, and had lower educational degrees than women (results not shown).

### 3.2. Hypothesis 1—Age-Differences in OSH Service Access and Awareness

A pronounced age gradient was found for all six indicators of access to OSH services. Young people worked less frequently in companies that provided access to OSH services, such as an occupational health physician, a safety and health manager, a workers’ representative for OSH, or safety training (Table 2). Accordingly, young workers reported less awareness of OSH issues than workers aged 30 years and older. For instance, they were less aware of basic regulatory frameworks, personal responsibility for health and safety, and emergency procedures. Age differences were significant in the unadjusted models and in the models adjusted for socio-demographic variables. Additional adjustment for employment conditions, however, resulted in a considerable reduction of the odds ratios for almost all indicators of access to OSH services. Adjustment had less of an effect on the estimators for age-differences in relation to awareness. Gender specific analyses revealed that the relative differences by age were largely similar for men and women, but women in all age groups had less access to OSH services than men (results not shown).

### 3.3. Hypothesis 2—Impact of Employment Conditions on Age-Differences

To further investigate the impact of employment conditions on age-differences in OSH access, we estimated additional regression models where we included each employment-related variable separately and compared the observed changes of age-differences before and after adjustment. The rationale for this procedure was to identify the single factors with the largest relative impact on the effect estimator for the association between age and the respective OSH indicator. Adjustment started with the employment condition with the lowest relative impact. Table 3 shows detailed results for three indicators where we observed the largest reduction in the odds ratio for age after adjusting for employment related variables (see Table 2). Stepwise adjustment revealed that the reduction in odds ratios for the association between age and OSH indicators was largely driven by the two indicators of company size and non-permanent employment contracts. In the case of the indicator, ‘no health and safety manager’, however, even after adjustment for all covariates, the odds ratios for age were still significantly elevated.

## 4. Discussion

The results of this study suggest that young workers have less access to occupational safety and health services and are less aware of OSH issues compared with older workers (Hypothesis 1). Differences were mostly medium to high, and were consistent for men and women. Results also indicate that that the precarious employment situation of younger workers in times of high youth unemployment contributes to the differences between younger and older employees in this study (Hypothesis 2). Temporary employment and employment in small companies were, in particular, related to lower OSH access and awareness in young workers.

There are only a few empirical studies that have examined how the age of an employee is associated with OSH service provision and quality, and our findings align with these previous findings. Breslin and colleagues (2005) reported that an adjustment for the type of occupation significantly reduced effect estimates for the association between work injury and younger employees in a large study of Canadian workers. They concluded that higher injury risks in young workers are due to their selection into more dangerous jobs in smaller-companies with less efficient systems of OSH protection. In a previous analysis presented by our working group using data from the European Working Conditions Survey, we found that young workers were less well informed about safety issues than older workers [10]. This statistical association was substantially weakened when employment related factors (e.g., economic sector, company size, or type of contract) were considered.

The finding that a selection of young people with precarious employment in occupations with less developed OSH management systems explained disadvantages in OSH protection points to the importance of considering structural factors in OSH research and practice [15]. Emphasis was placed on the improvement of existing OSH training programs, including strategies on how to cope with the lower experience and awareness of young people in this context [4,11]. This focus is undoubtedly important, however, structural disadvantages in access to different types of services and a related lower individual awareness deserves a broader approach to improve equitable access to OSH training. Evidence from previous studies underlines that precarious work is systematically associated with lower standards of OSH protection due to several reasons, such as a lower investment into the training of non-permanent workers or a missing voice of young workers [2,14,26,31,32,33]. It is, therefore, necessary to address the questions of how to better reach marginalised groups among the young workforce on a structural level [31]. This may include policies at different levels, such as labour policies aiming to improve employment conditions, improvements of OSH regulations relevant for young workers, promotion of participation and representation of young workers in planning and implementing OSH management, and a stricter control of compliance with safety regulations in those sectors with high proportions of young, precarious workers by the respective inspectorates [1,6,42,43,44]. Moreover, awareness about the specific needs of young workers must be promoted among employers as the engagement of the employer is crucial for guaranteeing a full coverage OSH system [37]. This includes a close and participative collaboration between employers, employees, and their representatives, like trade unions [45]. It is also well known that many small companies do not have the resources to implement high standard OSH services [46,47]. Supporting these companies to implement basic OSH services would likely improve safety and health among young people who are disproportionally employed in small businesses.

However, alternative explanations for age-differences have to be considered and it should also be pointed out that employment conditions did not fully account for age-differences in our study. A lack of awareness can also be driven by the comparably good health status of young persons, which may blur the need for informing oneself about OSH issues. Many age-related diseases and the impact of repeated exposure to occupational stressors are not necessarily factors that affect younger workers [48]. Accordingly, the general health status of young workers is better than for older workers [36,41]. It has also been observed that age segregation itself may foster a risk taking safety culture. As young people tend to take safety and health less seriously than older and more experienced workers, dysfunctional norms can manifest in workplaces with a high share of young people, such as those in food and beverage occupations [12,41]. Concerning access, seniority could also confound the association between age and access. The longer a person stays in a workplace, the higher the chance of encountering OSH services and of receiving safety training.

Methodological limitations must be considered when interpreting our findings. The quantitative design of the study does not allow us to explore the underlying reasons for differences in OSH service access and awareness in more detail as information about the particular workplaces was limited (e.g., presence of an OSH management plan was unknown). Next, the variables were all self-reported and based on single items. While this is not a problem in the case of awareness where the subjective view is of interest, it is possible that the reports about access to OSH services were biased and did not reflect true differences in objective access. Thus, we cannot conclude whether services were not provided by the employers or if young workers were not aware of their presence. It is evident at this point that additional (qualitative) research is needed to further explore the underlying reasons of age-differences from the employee’s perspective. Another source of possible bias is an underrepresentation of young people working in particular precarious conditions, such as workers without an employment contract. It is likely that their participation rate in a survey aiming at formally employed persons was low. Also, data is limited to Italy, and results are possibly not transferable to other countries. Another issue is gender differences. Age-dependency of awareness and access were comparable for male and female workers. However, this does not imply that, on a general level, no gender differences exist. For instance, access to OSH services may differ between men and women irrespective of age. Lastly, to investigate our second research question in more detail, future investigations may investigate the direct and indirect effects of age on OSH outcomes with more refined statistical methods, for example, structural equation modelling [49].

Limitations are balanced by a number of strengths. First, we had information about many indicators for several core OSH services and for different aspects of awareness of OSH. This allowed us to demonstrate the consistency of findings across different dimensions. Second, although the number of young workers in this study was limited due to the low labour market participation of the population under 30 years, the sample was large enough to study age-variance in relation to a number of important employment conditions.

## 5. Conclusions

To conclude, occupational safety and health (OSH) protection is an essential pillar of primary and secondary prevention. Access to and the quality of occupational safety and health protection for young workers does not appear equivalent with older workers, and, therefore, constitutes an important challenge for OSH systems. Young workers are not only more vulnerable in terms of injury risk and other health consequences related to their work, but may potentially also exhibit a lower awareness of OSH issues throughout their later working career.

## Figures and Tables

**Table 1:** Description of the analytical sample and main study variables (*n* = 8000; INSuLa 1 survey 2014; numbers and column percentage (%) or mean ± standard deviation).

Participant Characteristics		Age Groups				*p* *
		19–29 Years (*n* = 732)		30–64 Years (*n* = 7268)		
		Number	% or Mean	Number	% or Mean	
**Gender**	Male	445	60.8%	3860	53.1%	<0.001
	Female	287	39.2%	3408	46.9%	
**Age**	Mean	24.0 ± 2.6		44.4 ± 8.5		<0.001
**Education ^1^**	No/Primary	195	27.5%	2266	31.6%	0.002
	Secondary	395	55.6%	3498	48.7%	
	Tertiary	121	17.0%	1417	19.7%	
**Occupation.**	Non-manual	232	31.7%	3759	51,7%	<0.001
**Position**	Manual	438	59.8%	3306	45.5%	
	Trainee & other	62	8.5%	203	2.8%	
**Contract ^2^**	Permanent	421	58.5%	6323	87.4%	<0.001
	Non-permanent	299	41.5%	909	12.5%	
**Working hours**	Mean hours per week	37.5 + 9.7		36.5 + 9.1		0.003
**Sector of Economy**	Agriculture, hunting, fishing	17	2.3%	169	2.3%	<0.001
	Manufacturing/industry/energy	152	20.7%	1704	23.4%	
	Construction	71	9.7%	373	5.1%	
	Wholesale and retail trade	134	18.3%	838	11.5%	
	Hotels, bars, and restaurants	75	10.2%	366	5.0%	
	Transportation and storage	24	3.3%	402	5.5%	
	Information and communication	21	2.9%	187	2.6%	
	Business services, renting professional activities, finance, real estate, travel	114	15.6%	824	11.3%	
	Health and social work	28	3.8%	670	9.2%	
	Education	12	1.6%	681	9.4%	
	Public administration	5	0.7%	495	6.8%	
	Other community and personal services	80	10.9%	558	7.7%	
**Company Size ^3^**	Small (1–9 pers.)	237	34.2%	1079	15.5%	<0.001
	Medium (10–49 pers.)	167	24.1%	1448	20.8%	
	Large (50–249 pers.)	118	17.1%	1569	22.6%	
	Very large (≥250 pers.)	170	24.6%	2849	41.0%	

* *p*-value for deviance comparing age groups; chi ^2^-test or Mann-Whitney-U test; ^1^ excluding 108 participants who refused to answer; ^2^ excluding 48 participants unable to indicate their type of contract; ^3^ excluding 363 participants unable to quantify their company’s size.

**Table 2:** Occupational safety and health (OSH) service access and awareness by age groups; prevalence rates (numbers) and results from logistic regression analyses (odds ratios (OR) [95% confidence interval]).

OSH indictors	Age (*n* = 7612) ^1^ <30 y. (*n* = 732) 30+ y. (*n* = 7268)	Prevalence (Number)	Model 1, Unadjusted OR [95% CI]	Model 2 Adjustment Set 1 ^2^	Model 3 Adjustment Set 2 ^3^
**Access**					
No health and	<30 years	34.3 (251)	1.80 [1.52–2.13]	1.88 [1.58–2.23]	1.24 [1.03–1.5]
safety manager	30+ years	23.2 (1686)	reference	reference	reference
No occupation.	<30 years	34.0 (249)	1.36 [1.15–1.61]	1.44 [1.21–1.70]	1.03 [0.85–1.25]
physician	30+ years	28,1 (2042)	reference	reference	reference
No examination	<30 years	10.4 (50)	1.41 [1.02–1.94]	1.43 [1.04–1.98]	1.17 [0.82–1.68]
by occupational physician ^#^	30+ years	7.8 (406)	reference	reference	reference
No OSH	<30 years	39.9 (292)	2.23 [1.88–2.62]	2.35 [1.99–2.78]	1.44 [1.20–1.74]
Workers representative	30+ years	23.9 (1740)	reference	reference	reference
No OSH	<30 years	31.4 (230)	1.60 [1.35–1.90]	1.65 [1.39–1.96]	1.20 [0.99–1.45]
training	30+ years	23.1 (1682)	reference	reference	reference
No OSH	<30 years	16.1 (118)	1.52 [1.22–1.88]	1.56 [1.26–1.95]	1.08 [0.85–1.38]
information	30+ years	11.8 (860)	reference	reference	reference
**Awareness**					
No awareness	<30 years	4.1 (30)	1.97 [1.27–3.05]	2.22 [1.39–3.38]	1.90 [1.18–3.06]
of legal OSH framework	30+ years	2.2 (160)	reference	reference	reference
No awareness of	<30 years	23.5 (172)	1.49 [1.23–1.80]	1.55 [1.29–1.88]	1.21 [0.98–1.48]
fire & emergency procedures	30+ years	17.7 (1286)	reference	reference	reference
No awareness	<30 years	37.2 (272)	1.20 [1.02–1.41]	1.23 [1.05–1.45]	1.16 [0.97–1.38]
of first aid procedures	30+ years	34.0 (2472)	reference	reference	reference
No awareness	<30 years	6.1 (45)	1.78 [1.25–2.54]	1.80 [1.26–2.57]	1.56 [1.06–2.29]
of personal OSH responsibility	30+ years	3.5 (254)	reference	reference	reference
Not collected	<30 years	53.6 (392)	1.17 [0.99–1.37]	1.21 [1.03–1.41]	1.18 [0.99–1.40]
OSH information autonomously	30+ years	50.1 (3638)	reference	reference	reference

^1^ excluding 388 cases with missing values on one or more variables; ^2^ set 1 adjusted for sex, education; ^3^ set 2 adjusted for sex, education, occupational position, type of contract, working hours, economic sector, company size; ^#^ excluding participants with no access to an occupational physician (*n* = 5709).

**Table 3:** Age-differences in OSH service access after adjustment for employment conditions. Results of the logistic regression comparing young workers with workers aged 30–64 (reference), stepwise adjustment for covariates starting with a model adjusted for sex and education (odds ratios (OR) [95% confidence interval, 95%-CI]).

Adjustment	No Health and Safety Manager OR [95% CI] for Age (Young Compared to Older Workers)	No Occup. Physician OR [95% CI] for Age (Young Compared to Older Workers)	No OSH Information OR [95% CI] for Age (Young Compared to Older Workers)
Crude OR for age	1.80 [1.52–2.13]	1.36 [1.15–1.61]	1.52 [1.22–1.88]
OR for age after adjustment for:			
sex, education,	1.88 [1.59–2.23]	1.44 [1.22–1.70]	1.56 [1.26–1.95]
+ occupational position,	1.82 [1.53–2.16]	1.43 [1.21–1.71]	1.45 [1.16–1.82]
+ working time,	1.63 [1.54–2.18]	1.49 [1.26–1.79]	1.47 [1.18–1.83]
+ economic sector,	1.73 [1.45–2.07]	1.41 [1.17–1.69]	1.54 [1.22–1.94]
+ company size,	1.49 [1.24–1.79]	1.19 [0.99–1.44]	1.34 [1.06–1.69]
+ temporary contract	1.24 [1.03–1.50]	1.03 [0.85–1.25]	1.08 [0.85–1.38]
